# Esketamine ameliorates postoperative cognitive dysfunction aggravated by preoperative acute sleep deprivation in aged mice via the TLR4/NF-κB pathway

**DOI:** 10.3389/fnagi.2026.1798915

**Published:** 2026-03-23

**Authors:** Bo Han, Feifei Jin, Yana Zhong, Jinliang Teng

**Affiliations:** 1 Department of Anesthesiology, Hebei North University, Zhangjiakou, China; 2 College of Pharmacy, Hebei North University, Zhangjiakou, China; 3 Department of Pharmacy, The Second Affiliated Hospital of Hebei North University, Zhangjiakou, China; 4 Department of Anesthesiology, The First Affiliated Hospital of Hebei North University, Zhangjiakou, China

**Keywords:** esketamine, neuroinflammation, postoperative cognitive dysfunction, preoperative acute sleep deprivation, TLR4/NF-κB pathway

## Abstract

**Introduction:**

Postoperative cognitive dysfunction (POCD) is a common complication in elderly patients, and preoperative sleep deprivation may exacerbate its severity. Although earlier research has confirmed that esketamine can reduce the incidence of POCD, it remains unclear whether it is effective against POCD exacerbated by preoperative acute sleep deprivation. The present study is designed to examine the protective role and intrinsic mechanisms through which esketamine acts on POCD aggravated by pro operative acute sleep deprivation prior to surgery.

**Methods:**

The modified multiple platform method was employed to induce acute sleep deprivation, and the POCD model was established through left lateral lobectomy of the liver after 48 h of sleep deprivation. Esketamine was administered 30 min postoperatively. The Open Field Test and Morris Water Maze were utilized to assess postoperative cognitive alterations. Hippocampal pathology results were examined via hematoxylin–eosin staining; the concentrations of key inflammatory cytokines, namely interleukin-6 (IL-6), IL-1β, and tumor necrosis factor-*α* (TNF-α), were determined by enzyme-linked immunosorbent assay; the protein expression was analyzed using Western blotting and immunofluorescence analysis.

**Results:**

Esketamine administration post surgery significantly ameliorated cognitive deficits aggravated by preoperative acute sleep deprivation in aged mice. Concomitantly, esketamine downregulated the expression of TLR4, MyD88, and phosphorylated NF-κB p65 in hippocampal microglia, reduced hippocampal levels of IL-6, IL-1β, and TNF-α, and alleviated hippocampal tissue injury.

**Conclusion:**

Esketamine exerts neuroprotective effects against POCD exacerbated by preoperative acute sleep deprivation in aged mice by inhibiting the TLR4/NF-κB signaling pathway in hippocampal microglia.

## 1 Introduction

Following anesthesia and surgery, patients may develop postoperative cognitive dysfunction (POCD), a prevalent neurological complication. POCD, categorized as a member of the perioperative neurocognitive disorders family, is characterized by acute or persistent deficits in attention, concentration, learning, and memory that can persist for weeks or even several years (Evered et al., 2018; Zhao et al., 2024). POCD occurs more commonly in the elderly, and its incidence is influenced by factors such as underlying diseases, sleep disturbances, anesthesia, and surgery (Liu et al., 2023). Several investigations have indicated that sleep disorders affect approximately 30.6–41.2% of older adults living in community settings, whereas the incidence of preoperative sleep disturbances among hospitalized surgical patients can be as high as 60–86% (Wesselius et al., 2018; Butris et al., 2023). Moreover, multiple preclinical and clinical investigations have demonstrated that sleep disturbance prior to surgery is a prominent risk factor for POCD (Campbell and Figueiro, 2024; Ni et al., 2019; Li et al., 2025; Wang et al., 2021). According to a clinical study, sleep deprivation occurring during hospitalization due to pain, noise, illumination, anxiety, and medical procedures could elevate the vulnerability of elderly patients to POCD (Stewart and Arora, 2022). A rodent study also demonstrated that sleep deprivation prior to surgery aggravated cognitive impairment after surgery in aged mice (Li et al., 2025).

Currently, there are no definitive treatments for POCD in clinical practice. However, published reports have indicated that perioperative pharmacological interventions, including dexmedetomidine, lidocaine, and esketamine, may help prevent its occurrence (Chen et al., 2024a; Geng et al., 2023; Han et al., 2023). Notably, esketamine, the enantiomer of racemic ketamine, functions as a potent and selective antagonist at the N-methyl-D-aspartate (NMDA) receptor. It displays a stronger affinity toward NMDA receptors and certain opioid receptor subtypes compared to racemic ketamine (Mion and Himmelseher, 2024). While providing significant sedative and analgesic effects, it shows a relatively lower incidence of associated adverse reactions (such as psychiatric symptoms) (Nikayin et al., 2022). Preclinical and clinical studies have demonstrated esketamine’s efficacy in ameliorating POCD (Zhou et al., 2023; Zhang et al., 2023; Wang et al., 2024; Li et al., 2022). Despite these findings, the existing research is predominantly derived from models of surgical trauma in isolation, leaving a critical translational gap regarding its efficacy against POCD exacerbated by common clinical comorbidities. Notably, preoperative sleep deprivation is a highly prevalent yet understudied risk factor that may limit the prophylactic efficacy of esketamine. Therefore, it remains unknown whether esketamine can improve postoperative cognitive impairment that has been exacerbated by preoperative sleep deprivation. This study aims to investigate whether esketamine intervention alleviates POCD aggravated by preoperative acute sleep deprivation. Our results would help to elucidate esketamine’s potential role and neuroprotective mechanisms in POCD exacerbated by preoperative acute sleep deprivation.

## 2 Materials and methods

### 2.1 Animals

This study utilized 18-month-old male C57BL/6 mice (weighing 28–32 g), purchased from Sibeifu Biotechnology Co., Ltd. (Beijing, China). Mice were housed with 4–5 per cage in a specific pathogen-free facility and maintained under conditions where temperature was regulated at 23 ± 2 °C, humidity at 45–55%, and a 12-h light/dark cycle was implemented (lights from 9 a.m. to 9 p.m.). Animal experiments were approved by the Animal Ethics Committee of Hebei North University (Hebei, China) and conformed to the principles governing the use and care of experimental animals outlined in the US guidelines (NIH publication #85-23, revised 2011).

### 2.2 Experimental design

**Figure 1:**
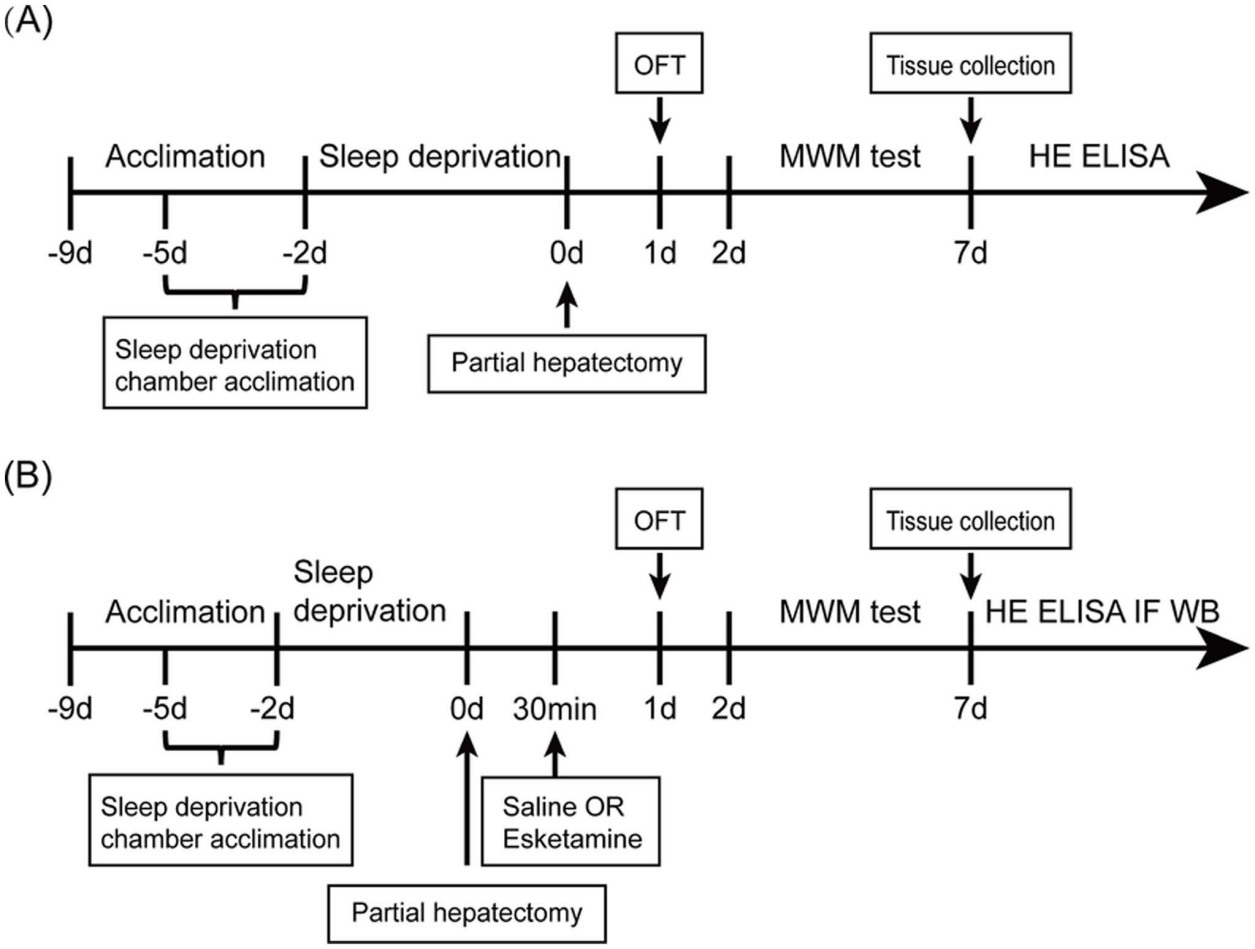
Study design. **(A)** Schematic timeline of experiment 1. **(B)** Schematic timeline of experiment 2.

To investigate the impact of preoperative acute sleep deprivation on postoperative cognitive function, we conducted experiment 1, in which the mice were randomly allocated into four experimental groups: (a) sham group, (b) surgery (Sur) group, (c) sleep deprivation (Sd) group, (d) sleep deprivation + surgery (Sd + Sur) group. The mice were subjected to partial hepatectomy to induce cognitive dysfunction following sleep deprivation. Twenty-four hours after the operation, behavioral assessments were performed. (Figure 1A).

To investigate the impact of esketamine on POCD exacerbated by preoperative acute sleep deprivation in aged mice, we conducted experiment 2. The mice with cognitive dysfunction exacerbation were randomly assigned to two groups: (a) control (Con) group and (b) esketamine treatment (Esk) group. The mice in the Esk group administered esketamine(Hengrui, Jiangsu, China) via intraperitoneal injection at a dose of 10 mg/kg, 30 min after surgery. The Con group was injected with an equal volume of 0.9% sterile saline (Figure 1B). Based on the doses documented in the research (Masaki et al., 2019; Wen et al., 2024), we selected 10 mg/kg of esketamine to explore the effects on our murine model.

At the end of the experiment, following the exclusion of animals that died or were removed during the experimental procedures, eight mice were ultimately included in each group (*n* = 8/group).

### 2.3 Sleep deprivation and partial hepatectomy

Sleep deprivation was achieved in mice by employing a modified multiple-platform approach (Li et al., 2024). Briefly, the apparatus was set up by placing 12 cylindrical platforms (3 cm in diameter, 6 cm in height) inside a polypropylene tank (50 × 40 × 25 cm). Water was then added to a level approximately 1 cm below the platform surfaces. Access to food and water was provided via a metal grid suspended above the water. During the last 3 days of the acclimation period, to allow for habituation, mice in the sleep-deprivation group were assigned to undergo daily 1-h exposures (10–11 a.m.) to the deprivation chamber prior to the formal protocol. Each chamber accommodated six mice, which could move freely across the platforms. During REM sleep, muscle atonia causes the head to contact the water surface, thereby preventing sustained sleep. A continuous 48-h deprivation period was implemented in the current experiment.

Partial hepatectomy was performed 6 h after sleep deprivation. Anesthesia was maintained with isoflurane, employing an induction concentration of 3.5% followed by a maintenance level of 0.8–1.5%. The surgical site was prepared by removing the fur and performing sequential disinfection with iodophor and 70% ethanol. An approximately 1.5-cm longitudinal incision was made below the xiphoid process to expose the liver. The left lateral lobe was resected with surgical silk suture after ligation of its vascular pedicle. The abdominal wall (muscle layer and skin) was closed using sterile 4–0 sutures, followed by iodophor disinfection. All of the procedures were performed under aseptic conditions. The entire surgical procedure was completed in approximately 30 min. The sham group and the Sd group underwent anesthesia, shaving, and disinfection. A laparotomy was performed followed by suture without liver lobe resection; the duration of anesthesia was consistent with that of the Sur group.

### 2.4 Open field test

The open field test (OFT) was conducted using a cubic arena (40 cm × 40 cm × 40 cm) constructed of white opaque material. OFT was conducted according to the method described in the experiment by Wu et al. (2024). On postoperative day 1, individual mice were introduced into the center of the arena, facing away from the observer, and allowed 5 min of unrestricted exploration commencing from the point of release. During inter-trial intervals, the arena was thoroughly wiped with ethanol to remove olfactory cues. A video-based tracking system was used to automatically capture the trajectory and quantify ambulatory metrics, including total distance moved, the average speed of locomotion, and time spent in the central area.

### 2.5 Morris water Maze test

With minor modifications, the Morris water maze (MWM) test was conducted in our study, which included opaque water maintained at 22° ± 1 °C and a platform submerged 1.0 cm below the water surface, as described in the study by Chen et al. (2024a). The water maze was partitioned into four equal quadrants, with the hidden platform positioned in one of them. Throughout the training trials, we gently placed the mice into the water from separate quadrants; the animals were then allowed to swim freely until they identified the hidden platform. A 90-s time limit was imposed for platform localization, and subsequent to finding it, the mice remained on the platform for 15 s. Alternatively, during the training phase, any mice unable to find the platform were directed to it and allowed to stay on it for 15 s, with their latency recorded as 90 s. Training was conducted four times daily for 5 successive days. In the spatial probe, the platform was withdrawn, and each mouse was placed into the quadrant farthest from the platform’s original position. The frequency of platform crossings and the duration of time spent in the target quadrant were documented.

### 2.6 Hematoxylin–eosin staining

After deep anesthesia with sodium pentobarbital (2%, 40 mg/kg), the mice received transcardial perfusion with physiological saline followed by 4% paraformaldehyde. The whole brains were removed and post-fixed in 4% paraformaldehyde for 48 h, followed by paraffin embedding. Coronal sections containing the hippocampus were fabricated and subjected to hematoxylin–eosin (H&E) staining, followed by observation of pathological lesions under a light microscope.

### 2.7 Western blot

Western blotting was employed to determine the expression levels of TLR4, MyD88, and NF-κB. On ice, hippocampal tissues were cut into small pieces and homogenized using RIPA lysis buffer (G2002-100ML; Servicebio, Wuhan, China) containing a protease inhibitor, followed by lysis for 15 min at 4 °C. The samples were centrifuged at 12,000 rpm for 15 min at 4 °C, after which the supernatant was obtained, and a BCA kit (G2026-200 T; Servicebio) was employed to assess the protein concentration. Sodium dodecyl sulfate-polyacrylamide gel electrophoresis (SDS-PAGE) was first conducted, after which the proteins were transferred onto a polyvinylidene fluoride (PVDF) membrane that had been pre-activated with methanol, followed by blocking with a blocking buffer at room temperature for 30 min. Following this step, overnight incubation at 4 °C was performed on them with the following diluted primary antibodies: TLR4 (GB11519, 1:1000; Servicebio), MyD88 (GB111554, 1:1000; Servicebio), p-NF-κB p65 (3,033 s, 1:1000; Cell Signaling Technology, Danvers, MA, USA), and GAPDH (GB15004, 1:1000; Servicebio). Post primary incubation, the membranes were subjected to three washes with Tris-buffered saline with Tween (TBST), 5 min per wash, prior to incubation with horseradish peroxidase-labeled goat anti-rabbit secondary antibody (GB23303, 1:5000; Servicebio) at room temperature for 30 min; additional washing with TBST was then carried out three times. Freshly prepared electrochemiluminescence solution (G2020-25ML; Servicebio) was applied evenly onto the membranes, which were then incubated for 1 min at room temperature while protected from light. Chemiluminescence exposure was performed in a darkroom, with exposure time adjusted according to signal intensity. Finally, the films were scanned, and the optical density of the target bands was analyzed using ImageJ software.

### 2.8 Enzyme-linked immunosorbent assay

Quantification of IL-6, IL-1β, and TNF-*α* levels was performed on hippocampal sections isolated from mice. We sonicated hippocampal samples in RIPA buffer containing protease inhibitors. The mixtures were then centrifuged at 12,000 rpm for 20 min at 4 °C, and the resulting supernatants were retained for ELISA detection. The expression levels of IL-6 (JI-ICHI, YZ-091206M), IL-1β (JI-ICHI, YZ-203063M), and TNF-α (JI-ICHI, YZ-201406M) were measured in accordance with the instructions provided in the corresponding ELISA kits.

### 2.9 Immunofluorescence

Dehydrated, paraffin-embedded brain tissue samples were sectioned serially; they were then deparaffinized and rehydrated in sequential washes using xylene, absolute ethanol, gradient ethanol, and finally distilled water. After completing antigen retrieval, the tissue sections were subjected to fixation, followed by a 30-min blocking step with 3% BSA (GC305010; Servicebio). Following this step, overnight incubation at 4 °C was performed on the sections using the primary antibodies anti-Iba1 (GB113502; 1:5000; Servicebio) and anti-p-p65 (AF2006; 1:2000; Affinity Biosciences, Changzhou, China). Following several washes, the sections were subjected to incubation with a fluorescent secondary antibody (GB23303; 1:500; Servicebio) and the sections were counterstained using DAPI (G1012; Servicebio) to enhance nuclei visualization. Finally, the images were captured using fluorescence microscopy.

### 2.10 Statistical analyses

Data are shown as mean ± SD throughout the study. The normality of distribution and homogeneity of variance were assessed. An unpaired Student’s t-test was utilized for statistical comparison between the two independent groups. Comparisons among multiple groups were conducted using one-way analysis of variance (ANOVA) followed by Tukey’s *post hoc* test. The escape latency data recorded over the training days were analyzed using two-way repeated-measures ANOVA, with Tukey’s post hoc test applied for subsequent pairwise comparisons. All statistical analyses were performed using GraphPad Prism 9.0. A *p* < 0.05 is regarded as statistically significant.

## 3 Results

### 3.1 Preoperative acute sleep deprivation significantly aggravated postoperative cognitive dysfunction in aged mice

**Figure 2:**
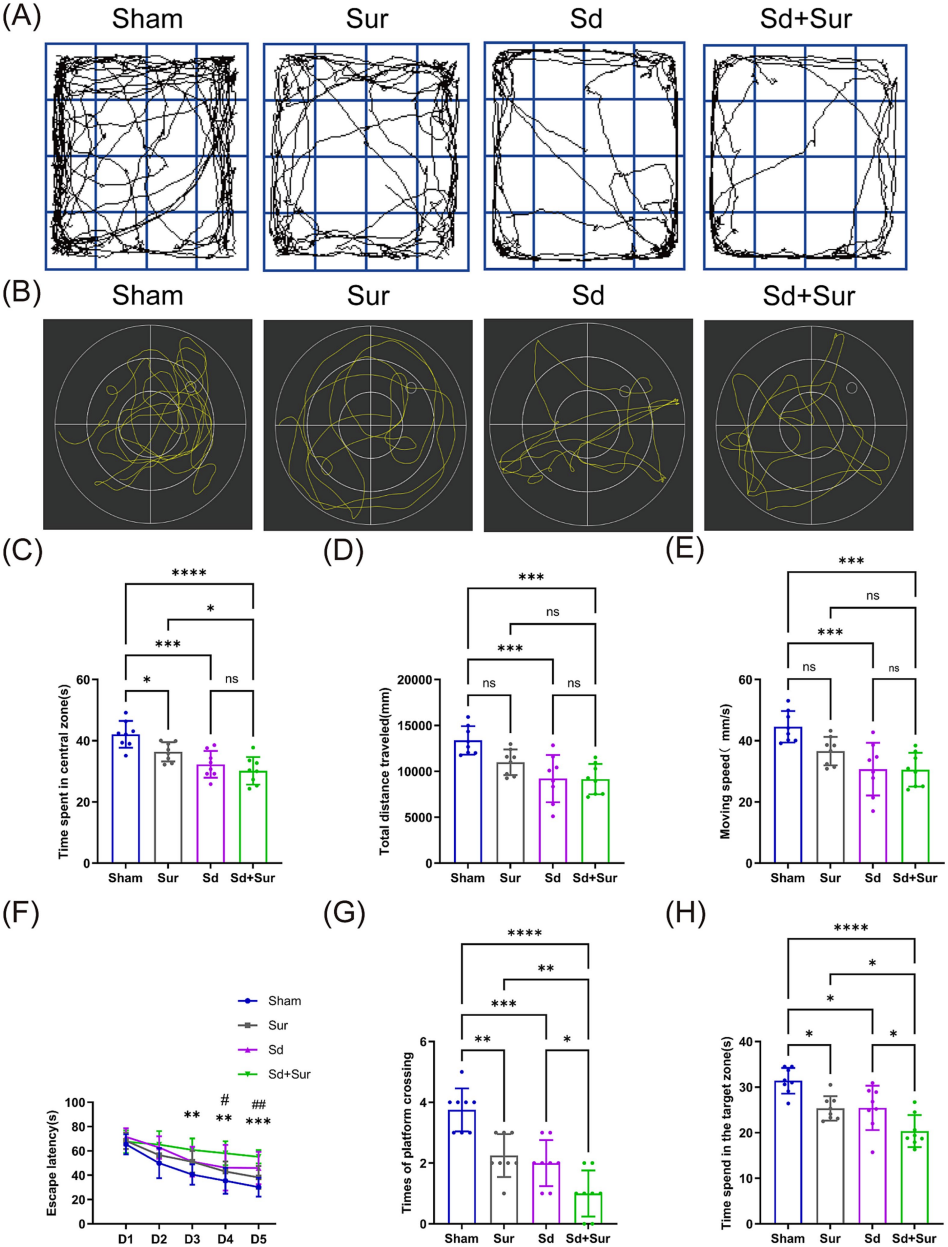
Preoperative acute sleep deprivation worsens cognitive impairment in aged mice with POCD. **(A)** Representative movement trajectories of each group in the OFT. **(B)** Representative swimming paths of the respective groups in the MWM test. **(C)** Time spent in the center zone by each group in the OFT. **(D)** Total distance traveled by each group in the OFT. **(E)** Moving speed by each group in the OFT. **(F)** Escape latency of each group during the acquisition phase of the MWM test (^**^p < 0.01, ^***^p < 0.001 vs. sham. ^#^*p* < 0.05, ^##^*p* < 0.01 vs. Sur). **(G)** Number of platform crossings for each group during the probe trial of the MWM test. **(H)** Time spent in the target quadrant by each group during the probe trial of the Morris water maze. Data are presented as mean ± SD values (*n* = 8 per group). NS: *p* > 0.05, ^*^p < 0.05, ^**^p < 0.01, ^***^p < 0.001, ^****^p < 0.0001.

To evaluate the impact of preoperative acute sleep deprivation on POCD, mice were subjected to partial hepatectomy after receiving sleep deprivation treatment to induce cognitive impairment, and the OFT and MWM were employed to analyze the changes in postoperative cognitive function. Figures 2A,B depict the track plots obtained from the OFT and MWM. OFT is a well-established assay in behavioral neuroscience, primarily assessing general locomotor activity while simultaneously providing insights into anxiety-related behaviors in experimental animals (Kraeuter et al., 2019). The discrimination index obtained from the OFT demonstrated that, in comparison with sham mice, those in the Sur group exhibited a shorter duration in the central zones, and that the time spent in the center by the Sd + Sur group was shorter than that by the Sur group (Figure 2C). Moreover, the Sd + Sur group exhibited reduced total distance traveled and moving speed in contrast to the Sur group, but no statistically significant differences were observed (Figures 2D,E).

The MWM has been previously employed to assess spatial memory and learning ability in experimental studies (Dinel et al., 2020). The MWM index showed that, as the number of training sessions increased, the escape latency of all mice significantly decreased, with the shortest escape time observed on day 5 after the completion of all training. In contrast to the Sur group, mice in the Sd + Sur group exhibited significantly longer escape latency on days 4 and 5 (Figure 2F). Furthermore, in comparison with the sham group, the Sur group exhibited a marked decrease in both the frequency of crossing the former platform location and the duration of exploration in the target quadrant. Relative to the Sur group, the Sd + Sur group showed significantly fewer platform crossings and a shorter duration spent in the target quadrant (Figures 2G,H). In conclusion, the behavioral results indicated that preoperative sleep deprivation exacerbated impairments in spatial learning and memory, as well as increased anxiety-like behavior in aged mice post surgery.

**Figure 3:**
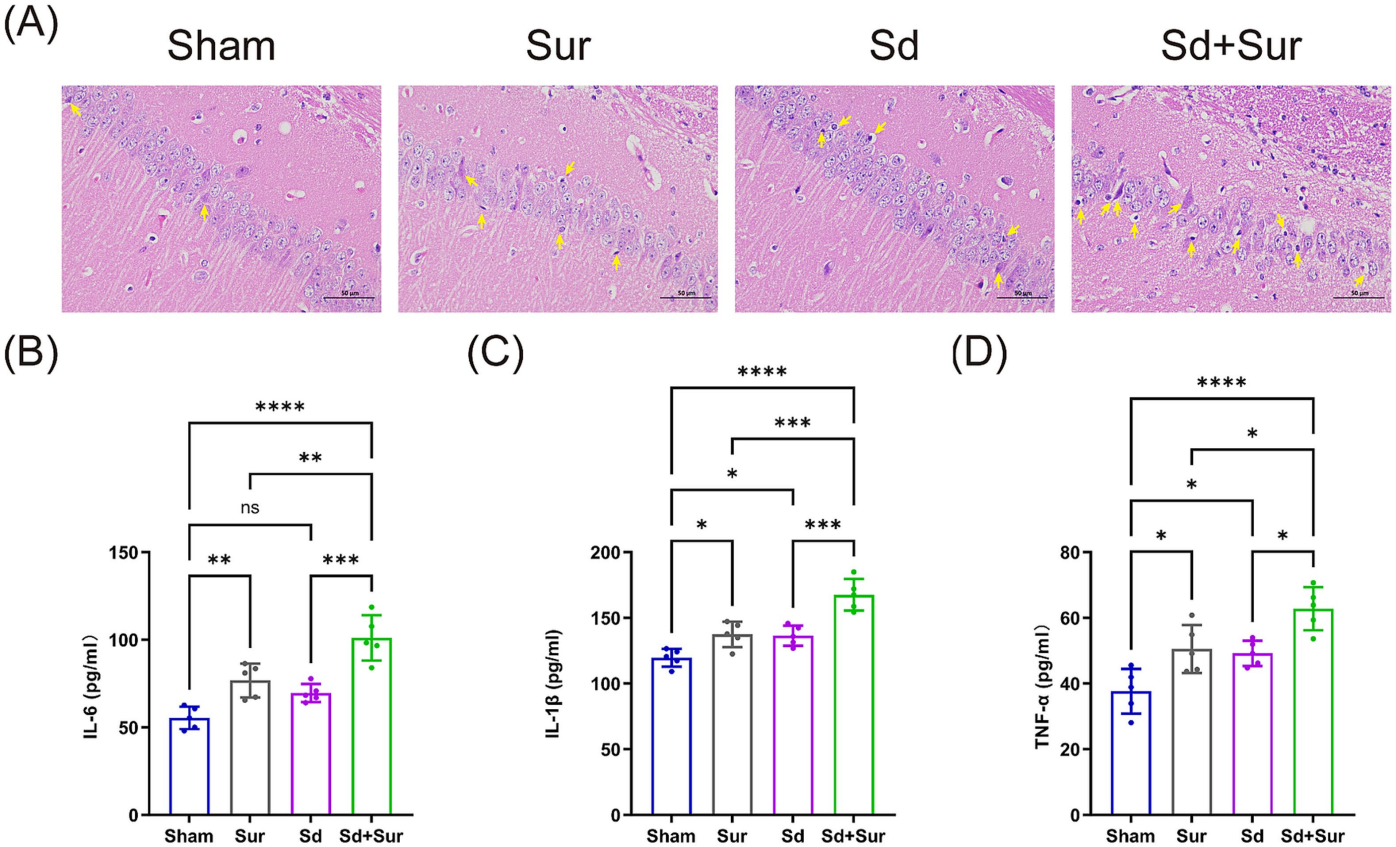
Preoperative acute sleep deprivation exacerbates surgery-induced hippocampal damage and neuroinflammation in aged mice. **(A)** Hippocampal H&E staining of each group (Scale, 50 μm). **(B)** Hippocampal levels of IL-6 measured by ELISA. **(C)** Hippocampal levels of IL-1β measured by ELISA. **(D)** Hippocampal levels of TNF-*α* measured by ELISA (*n* = 5 per group). Data are presented as mean ± SD values. NS: *p* > 0.05, ^*^p < 0.05, ^**^p < 0.01, ^***^p < 0.001, ^****^p < 0.0001.

We further observed hippocampal histology by H&E staining. We found that, in the sham group, the hippocampal CA1 region exhibited intact pyramidal cell layers, with densely packed and uniformly stained neurons. The nuclei were round and centrally located. The Sur group and Sd group displayed moderate neuronal degeneration in the CA1 region, characterized by shrunken cell bodies, pyknotic nuclei, and reduced neuronal density. Pathological changes were further exacerbated in the Sd + Sur group, which included a distinct decline in the quantity and density of neurons within the hippocampal CA1 area, accompanied by irregular arrangement and severe impairment of neurons (Figure 3A).

As reported in several studies, POCD is closely associated with central neuroinflammation (Liu et al., 2023). Therefore, we further determine the levels of pro-inflammatory cytokines in the hippocampus, including IL-6, IL-1β, and TNF-*α*. Compared with the sham group, the Sd and Sur groups exhibited elevated levels of the proinflammatory cytokines IL-6, IL-1β, and TNF-α. Additionally, compared to the Sur group, the Sd + Sur group showed significant increases in the levels of IL-6, IL-1β, and TNF-α (Figures 3B–D).

These results indicate that preoperative acute sleep deprivation exacerbates POCD induced by surgery, which is associated with hippocampal neuroinflammation and structure injury.

### 3.2 Esketamine relieved postoperative cognitive impairment aggravated by acute sleep deprivation in aged mice

**Figure 4:**
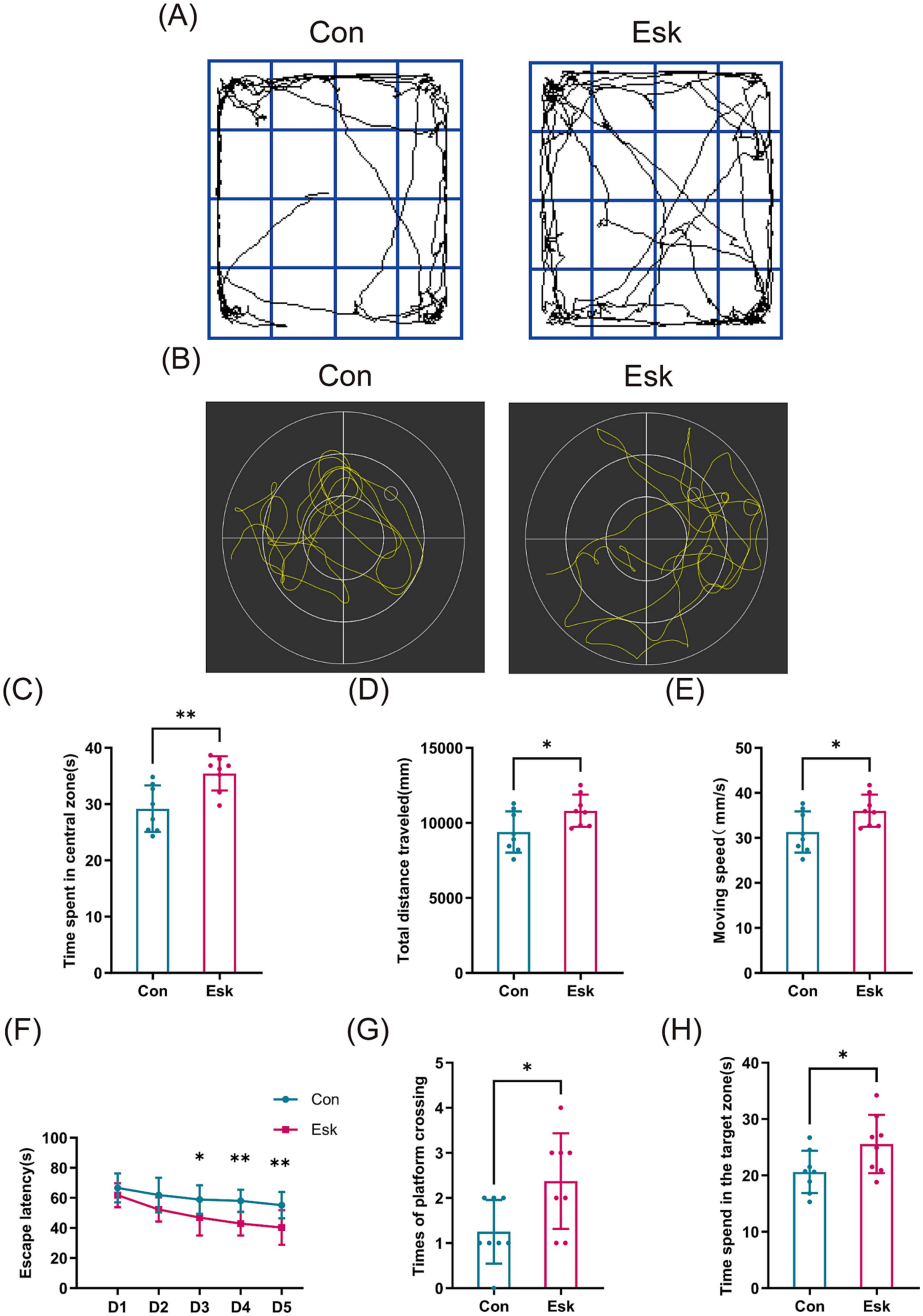
Esketamine ameliorates postoperative cognitive impairment in aged mice with preoperative acute sleep deprivation. **(A)** Representative movement trajectories of each group in the OFT. **(B)** Representative swimming paths of the respective groups in the MWM test. **(C)** Time spent in the center zone by each group in the OFT. **(D)** Total distance traveled by each group in the OFT. **(E)** Moving speed by each group in the OFT. **(F)** Escape latency of each group during the acquisition phase of the MWM test. **(G)** Number of platform crossings for each group during the probe trial of the MWM test. **(H)** Time spent in the target quadrant by each group during the probe trial of the MWM test. Data are presented as mean ± SD values (*n* = 8 per group). ^*^p < 0.05, ^**^p < 0.01.

With the aim of assessing the impacts of esketamine on cognitive impairment exacerbated by sleep deprivation, behavioral tests were performed using OFT and MWM. The path trajectory plots corresponding to the OFT and MWM are displayed in Figures 4A,B, respectively. In the OFT, as opposed to the control group, the esketamine-treated group presented notable increases in the duration spent in the center zone, total travel distance, and moving speed (Figures 4C–E). In the MWM test, compared with the control group, the mice in the treatment group showed a significant shortening of escape latency starting from day 3. Meanwhile, the esketamine-treated group showed statistically significant elevations in two key spatial memory indices: the frequency of platform crossings and the time residing in the target quadrant (Figures 4F–H).

**Figure 5:**
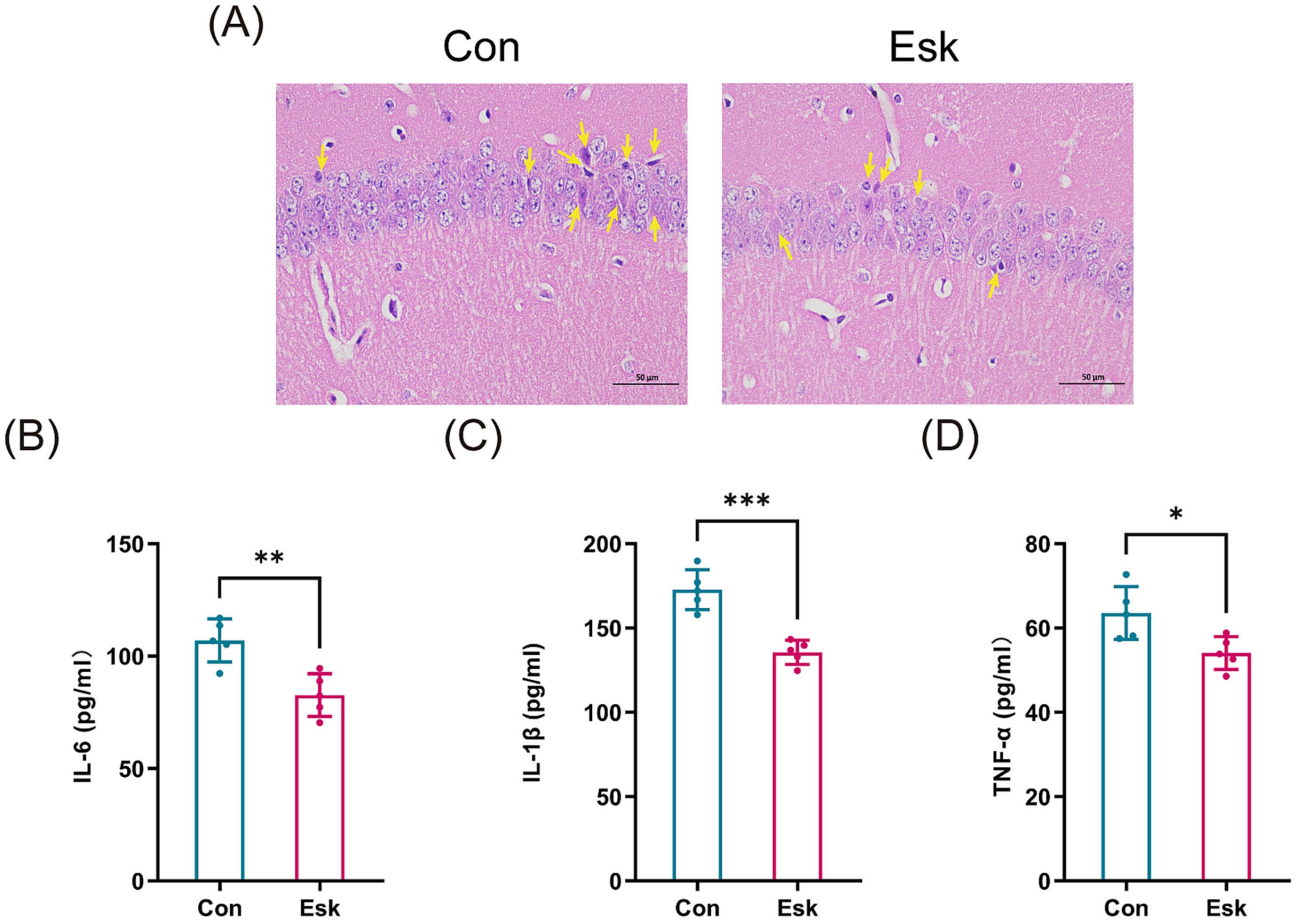
Esketamine ameliorates hippocampal neuroinflammation induced by surgery and preoperative acute sleep deprivation in aged mice. **(A)** Hippocampal H&E staining of each group (Scale, 50 μm). **(B)** Hippocampal levels of IL-6 measured by ELISA. **(C)** Hippocampal levels of IL-1β measured by ELISA. **(D)** Hippocampal levels of TNF-α measured by ELISA (*n* = 5 per group). Data are presented as mean ± SD values. ^*^p < 0.05, ^**^p < 0.01, ^***^p < 0.001.

We further observed hippocampal histology by H&E staining. In the hippocampal CA1 region of control mice, neurons were sparsely distributed, the tissue architecture was loose, and nucleoli had disappeared. However, the esketamine-treated group showed ameliorated damage to the hippocampal tissue structure and neurons (Figure 5A).

Since neuroinflammation is critically involved in the development and progression of POCD, we further measured the expression levels of hippocampal pro-inflammatory cytokines, including IL-6, IL-1β, and TNF-*α*. As shown by ELISA results, the levels of IL-6, IL-1β, and TNF-α in the hippocampus were notably decreased in the esketamine-treated group relative to the control group (Figures 5B–D).

These results showed that postoperative administration of esketamine effectively ameliorated POCD exacerbated by preoperative sleep deprivation in aged mice.

### 3.3 Esketamine suppressed the TLR4/NF-κB signaling pathway in hippocampal microglia

**Figure 6:**
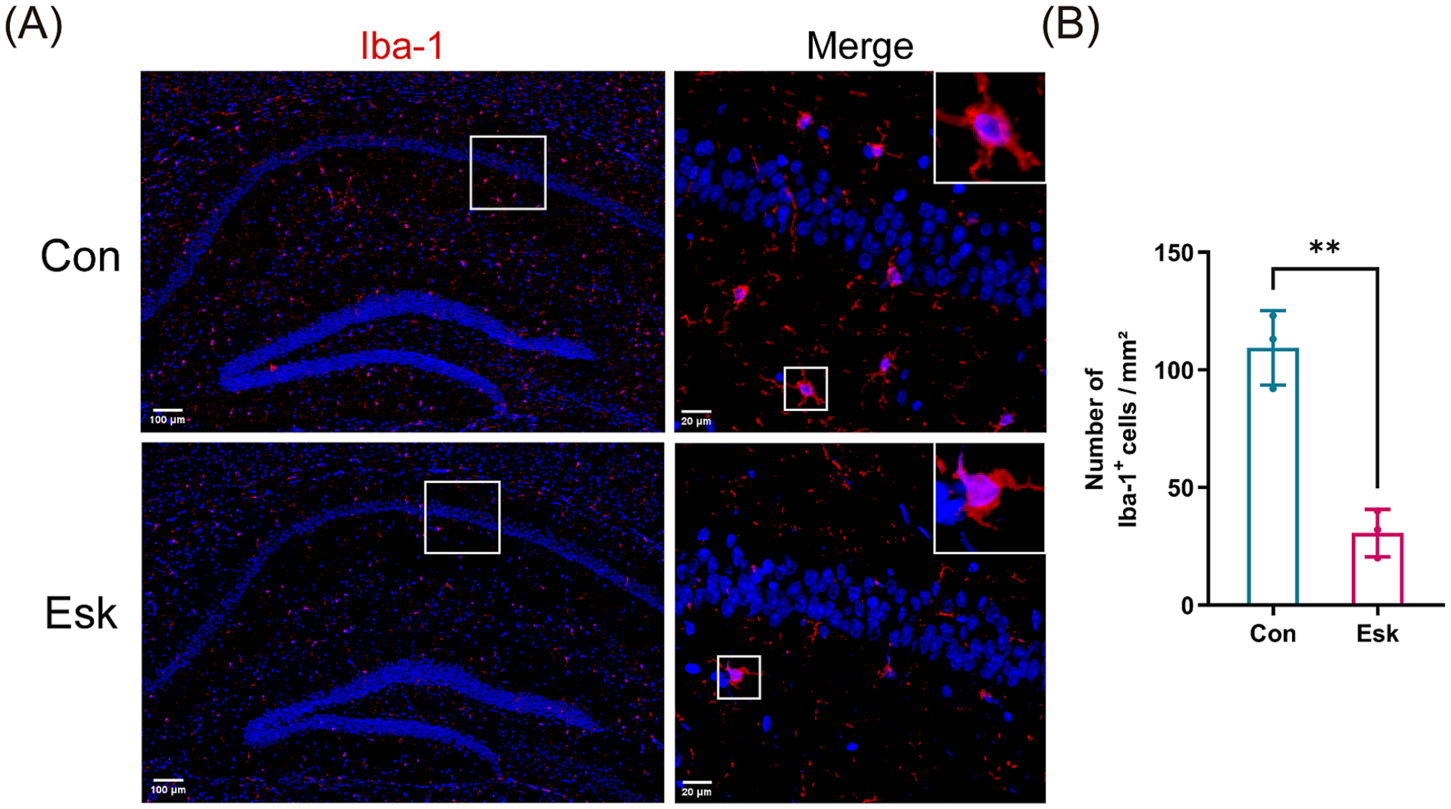
Esketamine inhibits microglial activation in the hippocampus of aged mice after preoperative acute sleep deprivation and surgery. **(A)** Representative photomicrographs of Iba-1 immunofluorescence in the hippocampus of each group. Scale, 100 μm and 20 μm. **(B)** Number of Iba-1 positive cells in the hippocampus of each group. Data are presented as mean ± SD values (*n* = 3 per group). ^**^p < 0.01.

**Figure 7:**
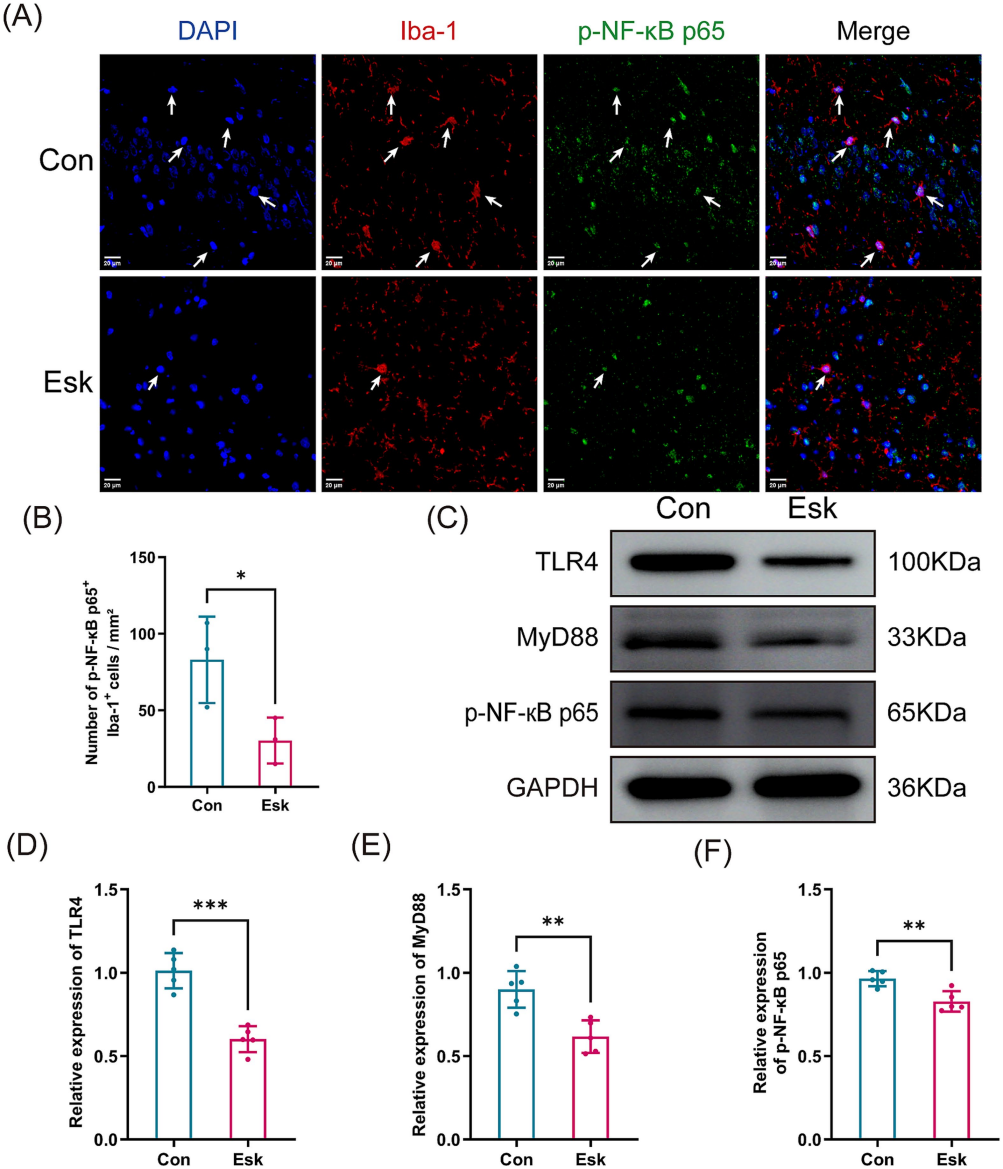
Esketamine suppressed the TLR4/NF-κB signaling pathway in the hippocampal microglia. **(A)** Representative dual immunofluorescent images of Iba-1/p-NF-κB p65 in the hippocampus of each group. Scale, 20 μm. **(B)** Percentage of Iba-1-positive cells that are p-NF-κB p65-positive in the hippocampus (*n* = 3 per group). **(C)** Representative western blot images of TLR4, MyD88, and p-NF-κB p65 in the hippocampus of each group. **(D–F)** Quantification of relative TLR4, MyD88, p-NF-κB p65 protein expression levels (*n* = 5 per group). Data are presented as mean ± SD values. ^**^p < 0.01, ^***^p < 0.001.

Microglia is a crucial source of inflammatory mediators in the hippocampus, and its activation is closely associated with postoperative cognitive impairment (Hu et al., 2024; Alam et al., 2018). To investigate the mechanism relative to the beneficial effect of esketamine, we carried out double-labeling immunofluorescence using antibodies against the microglia-specific marker Iba-1 and p-NF-κB p65, a key component of the TLR4/NF-κB axis. Immunofluorescence analysis revealed a significant reduction in the count of Iba-1 positive cells within the hippocampus of esketamine-treated mice relative to the control (Figures 6A,B). Additionally, a notable decrease in the number of p-NF-κB p65-positive cells within the Iba-1-positive cell population was observed in the esketamine-treated group (Figures 7A,B). To further validate these findings at the protein level, we performed the Western blot analysis. Consistent with the immunofluorescence data, the Western blot analysis revealed that, relative to the control group, the esketamine-treated group exhibited a significant decrease in the protein expression of TLR4, MyD88, and p-NF-κB p65 (Figures 7C–F). These results suggest that esketamine exerts an inhibitory effect on the activation of the TLR4/NF-κB signaling pathway in hippocampal microglia.

## 4 Discussion

In the present study, we found that postoperative administration of esketamine ameliorated POCD aggravated by preoperative acute sleep deprivation in aged mice, as demonstrated by the OFT and MWM test. We noted the beneficial effect of esketamine accompanied by damage alleviation of the hippocampal tissue structure and neurons and reduction of inflammatory mediators in the hippocampal tissue. We further found that esketamine exerted an inhibitory effect on both hippocampal microglial activation and the TLR4/NF-κB signaling pathway.

Sleep, which is one of the fundamental physiological needs in humans, plays a crucial role in the consolidation of learning and memory, along with in the maintenance of homeostasis within the brain microenvironment (Schaefer et al., 2019). Adequate sleep not only facilitates the restoration of physical energy but also optimizes brain function, strengthens memory retention, and enhances learning efficiency and creativity. Conversely, sleep insufficiency can lead to deficits in memory consolidation. With advancing age, the architecture of human sleep undergoes significant alterations, characterized by a reduction in overall sleep duration, prolonged sleep latency, and increased sleep fragmentation (Takamino et al., 2022). Acute sleep deprivation is defined as a state in which an individual experiences a complete or nearly complete lack of sleep over a short period, typically ranging from 24 to 72 h (Xu et al., 2025). Studies have demonstrated that chronic sleep deprivation reduces the synthesis of proteins associated with neuroplasticity in the hippocampal tissue, while acute sleep deprivation impairs functions of the medial temporal lobe, particularly those related to hippocampal-dependent learning and encoding processes (Fernandes et al., 2015; Krause et al., 2017). Furthermore, clinical evidence indicates that preoperative sleep deficiency constitutes a significant independent risk factor for postoperative cognitive decline (Takamino et al., 2022; Yang et al., 2023). In the present study, we employed the modified multiple platform technique to establish a sleep deprivation model in mice to simulate the preoperative sleep-disordered condition observed in clinical research involving patients. The behavioral experimental results confirmed that both preoperative sleep deprivation and surgical intervention resulted in further exacerbating POCD in aged mice. These results are consistent with the findings reported in the existing literature (Liu et al., 2025; Wu et al., 2023).

POCD is a common clinical complication after surgery. Although the adverse effects of POCD are widely recognized, its underlying pathogenesis and pathophysiology remain unclear. Current evidence suggests that the mechanisms of POCD primarily include neuroinflammation, impaired synaptic plasticity, aberrant post-translational modifications of tau protein, and mitochondrial dysfunction (Liu et al., 2023; Gao et al., 2021; Wang et al., 2023; Netto et al., 2018). During recent years, there has been widespread recognition that neuroinflammation serves as a pivotal factor in the occurrence and development of POCD (Liu et al., 2023; Yang et al., 2022). Multiple studies have indicated that surgical trauma can trigger a systemic inflammatory response and facilitate the secretion of proinflammatory cytokines (e.g., IL-6, IL-1β, and TNF-*α*) in the plasma, which compromises blood–brain barrier integrity, thereby allowing inflammatory mediators to enter the central nervous system (CNS) and induce neuroinflammation (Wen et al., 2020). Concurrently, anesthesia and surgery can activate microglia, contributing to the initiation and progression of neuroinflammation. Constituting the innate immune sentinel population within the CNS, microglia play an indispensable role in immune surveillance and monitoring (Fakhoury, 2018). Under pathological conditions, the activation of these cells triggers the release of numerous signaling molecules, including chemokines and cytokines, thereby propagating and intensifying neuroinflammation. If this activated state persists, it perpetuates a cascade of events including sustained CNS inflammation and injury to hippocampal neurons, which ultimately manifests as cognitive dysfunction and can progress to neurodegenerative diseases (Li et al., 2020). Importantly, sleep deprivation can induce neuroinflammation and exacerbate the neuropathological alterations triggered by surgery (Que et al., 2023). Research by Ni et al. demonstrated that preoperative sleep deprivation exacerbates the extent of surgery-induced microglial and astrocytic activation, as well as blood–brain barrier dysfunction (Ni et al., 2019). This not only aggravates neuroinflammation and neuronal damage but, more importantly, intensifies POCD in aged mice.

Based on the aforementioned mechanisms, reducing neuroinflammation through genetic regulation or pharmacological intervention is considered a potential strategy for mitigating cognitive impairment. Esketamine, the dextrorotatory enantiomer of ketamine, is being increasingly used in clinical practice because of its effective analgesic and sedative properties. Research has indicated that esketamine can modulate inflammatory responses by inhibiting the synthesis of pro-inflammatory cytokines, namely IL-6, IL-1β, and TNF-*α*, thereby attenuating postoperative neuroinflammation and preserving brain function (Han et al., 2023). A meta-analysis on esketamine and POCD in adults demonstrated that esketamine may reduce the risk of POCD by mitigating neuroinflammation and protecting neurons (Lin et al., 2024). Furthermore, several preclinical studies have suggested that esketamine could ameliorate POCD (Xu et al., 2024). However, evidence remains limited regarding whether esketamine can improve POCD exacerbated by preoperative sleep deprivation. The present study demonstrated that surgery elevated the concentrations of pro-inflammatory cytokines (IL-1β, IL-6, and TNF-α) in the hippocampus, and led to tissue disruption and neuronal damage. Preoperative sleep deprivation further exacerbated this surgery-induced neuroinflammation and neuronal injury, consequently leading to more severe learning and memory deficits. Notably, treatment with esketamine significantly inhibited the activation of hippocampal microglia and reduced pro-inflammatory cytokine levels. This clearly demonstrates that esketamine ameliorates neurocognitive impairment aggravated by preoperative sleep deprivation via suppressing the central nervous system inflammatory response.

The TLR4/NF-κB signaling pathway is a classical inflammatory regulation pathway and has attracted increasing attention in POCD research. Within the hippocampus, TLR4 is chiefly expressed on microglia and serves as a critical receptor for orchestrating their immune functions (Wang et al., 2022). After anesthesia and surgery, the ensuing systemic inflammatory response promotes the liberation of damage-associated molecular patterns (DAMPs). Upon recognition of these DAMPs by TLR4, the downstream MyD88/NF-κB signaling pathway is activated. A critical step in this activation is the phosphorylation of the NF-κB p65 subunit and its subsequent nuclear translocation, transforming it into its transcriptionally active form, p-p65. Therefore, in our experiments, the detection of p-p65 was chosen as a direct measure of this critical activation event, as it specifically reflects the NF-κB population that has translocated to the nucleus to drive pro-inflammatory gene expression. This process drives the activation of microglia and a robust secretion of pro-inflammatory factors, leading to neuroinflammation and subsequent cognitive deficits (Feng et al., 2017; Chen et al., 2024b). It has been reported that inhibiting TLR4 effectively attenuates microglial activation and neuroinflammation, thereby improving cognitive function (Chavan et al., 2012). In the present study, immunofluorescence double labeling and Western blot experiments further demonstrated that esketamine suppresses the expression levels of proteins involved in the TLR4/NF-κB signaling pathway and inhibits microglial activation in an aggravated POCD model in aged mice. These findings suggest that esketamine could ameliorate POCD aggravated by preoperative sleep deprivation in aged mice, and this beneficial effect is potentially mediated by its ability to suppress this signaling pathway, decrease the secretion of downstream inflammatory cytokines, and mitigate relevant neuroinflammatory reactions.

It should be noted that the current study is not without limitations. First, only the short-term effects of sleep deprivation on postoperative cognitive function were examined. Second, although preoperative sleep deprivation was found to exacerbate POCD, the underlying mechanisms were not investigated. Thus, elucidating the specific mechanisms through which preoperative sleep deprivation exacerbates POCD will be an important focus in future research.

In summary, these results revealed that esketamine alleviates POCD exacerbated by preoperative acute sleep deprivation in aged mice. The inhibition of the TLR4/NF-κB signaling pathway in hippocampal microglia constitutes the mechanistic basis of this protective effect, thereby mitigating neuroinflammation and conferring neuroprotection. These results highlight the therapeutic potential of esketamine for managing patients with POCD, particularly in the vulnerable aged populations with sleep disturbances.

## Data Availability

The original contributions presented in the study are included in the article/supplementary material, further inquiries can be directed to the corresponding author.
